# Poly(lysine) Dendrimers Form Complexes with siRNA and Provide Its Efficient Uptake by Myeloid Cells: Model Studies for Therapeutic Nucleic Acid Delivery

**DOI:** 10.3390/ijms21093138

**Published:** 2020-04-29

**Authors:** Michał Gorzkiewicz, Olga Kopeć, Anna Janaszewska, Małgorzata Konopka, Elżbieta Pędziwiatr-Werbicka, Irina I. Tarasenko, Valeriy V. Bezrodnyi, Igor M. Neelov, Barbara Klajnert-Maculewicz

**Affiliations:** 1 Department of General Biophysics, Faculty of Biology and Environmental Protection, University of Lodz, 141/143 Pomorska St., 90-236 Lodz, Poland; michal.gorzkiewicz@biol.uni.lodz.pl (M.G.); olga.kopec7611@gmail.com (O.K.); anna.janaszewska@biol.uni.lodz.pl (A.J.); gosiapicasa@gmail.com (M.K.); elzbieta.pedziwiatr@biol.uni.lodz.pl (E.P.-W.); 2 Institute of Macromolecular Compounds, Russian Academy of Sciences, Bolshoi Prospect 31, V.O., 199004 St. Petersburg, Russia; i.tarasenko@mail.ru; 3 Department of Physics, St. Petersburg State University (SPbSU), 7/9 Universitetskaya nab., 199034 St. Petersburg, Russia; v.v.bezrodniy@mail.ru; 4 Institute of Bioengineering, St. Petersburg National Research University of Information Technologies, Mechanics and Optics (ITMO University), Kronverkskiy pr. 49, 197101 St. Petersburg, Russia; i.neelov@mail.ru; 5 Leibniz-Institut für Polymerforschung Dresden e.V., 6 Hohe St., 01069 Dresden, Germany

**Keywords:** poly(lysine) dendrimers, siRNA, gene delivery, gene therapy, transfection

## Abstract

The disruption of the cellular pathways of protein biosynthesis through the mechanism of RNA interference has been recognized as a tool of great diagnostic and therapeutic significance. However, in order to fully exploit the potential of this phenomenon, efficient and safe carriers capable of overcoming extra- and intracellular barriers and delivering siRNA to the target cells are needed. Recently, attention has focused on the possibility of the application of multifunctional nanoparticles, dendrimers, as potential delivery devices for siRNA. The aim of the present work was to evaluate the formation of dendriplexes using novel poly(lysine) dendrimers (containing lysine and arginine or histidine residues in their structure), and to verify the hypothesis that the use of these polymers may allow an efficient method of siRNA transfer into the cells in vitro to be obtained. The fluorescence polarization studies, as well as zeta potential and hydrodynamic diameter measurements were used to characterize the dendrimer:siRNA complexes. The cytotoxicity of dendrimers and dendriplexes was evaluated with the resazurin-based assay. Using the flow cytometry technique, the efficiency of siRNA transport to the myeloid cells was determined. This approach allowed us to determine the properties and optimal molar ratios of dendrimer:siRNA complexes, as well as to demonstrate that poly(lysine) dendrimers may serve as efficient carriers of genetic material, being much more effective than the commercially available transfection agent Lipofectamine 2000. This outcome provides the basis for further research on the application of poly(lysine) dendrimers as carriers for nucleic acids in the field of gene therapy.

## 1. Introduction

RNA interference (RNAi) is a biological process in which RNA molecules induce specific inhibition of target gene expression. Its discovery provoked great enthusiasm in the scientific community, and subsequent studies on RNAi resulted in the transition from experimental technology to a powerful therapeutic tool. In 2010, the first case of systemic targeted delivery of short interfering RNA (siRNA) was reported, which gave a solid foundation for the clinical use of this type of genetic material [1]. In recent years, siRNA has become the basis for drug development due to the high level of specificity, limited side effects, and the ease of synthesis of therapeutic molecules [2].

However, before siRNA molecules reach their site of action in the cell cytoplasm, they have to conquer several obstacles, the number of which depends largely on the way of administration, the target tissue, as well as the physicochemical properties of the siRNA itself. The latter involve low stability, negative charge, and high structural stiffness, hampering the transport in body fluids, across cellular membranes, and between intracellular compartments [3]. What is more, siRNA upon entering the bloodstream easily undergoes enzymatic degradation by nucleases or rapid renal clearance [4].

In order to overcome the barriers limiting the transport of genetic material and exploit the therapeutic potential offered by the RNAi mechanism, effective delivery systems for siRNA molecules to their site of action are necessary. These systems, in addition to specific transport and transfection efficiency, should be able to protect siRNA against degradation by nucleolytic enzymes, prolong its blood circulation time, and release genetic material intracellularly, making it easily accessible for RNAi machinery, thus enabling effective silencing of the target gene [5].

In general, the carriers used to introduce nucleic acids into the cells can be divided into viral and non-viral systems. Vectors using genetically modified viruses are capable RNA delivery devices, offering, among others, long-term silencing of gene expression even after a single administration, efficient transfection, or expression of multiple copies of siRNA molecules. However, expensive production and side effects associated with random integration into the host genome (which can lead to the damage of important genes or activation of oncogenes), significant immunogenicity, and the possibility of the virus returning to its wild pathogenic type may limit the use of this group of carriers. Therefore, efforts have been made to obtain synthetic non-viral siRNA carriers, useful for both in vitro and in vivo applications [6,7].

Currently, the most commonly used non-viral siRNA carrier systems are based on nanomaterials. For this purpose, natural and synthetic cationic polymers are often applied [8], including highly branched dendrimers of a well-defined structure. These compounds enable electrostatic interactions with negatively charged siRNA molecules, providing the formation of stable complexes (dendriplexes) that are capable of intracellular delivery of nucleic acids and their protection against the activity of nucleases. What is more, surface groups of dendrimers may be subjected to modifications by functional moieties targeting them to specific locations. Numerous experiments have shown that dendrimers can be successfully used as carriers of various types of genetic material, including plasmids, single strands of DNA, oligonucleotides, and finally RNA molecules, ensuring their improved stability and prolonged blood half-life [9,10].

Most studies on the potential use of dendrimers as carriers for siRNA concern poly(amidoamine) (PAMAM), poly(propyleneimine) (PPI), and poly(lysine) dendrimers (PLL) belonging to the group of peptide dendrimers [10]. The latter are branched polymeric structures in which both the core and dendrons are composed mainly of amino acids connected by peptide bonds [11]. Peptide dendrimers are most often based on lysine, an amino acid that enables the generation of several branching points [12]. PLL dendrimers, due to their flexible structure and protein-like characteristics, such as good biocompatibility and water solubility, as well as high resistance to proteolytic digestion could be used in several biomedical applications. Their properties have been studied over the last few years both experimentally [13,14,15] and in computer simulation [16,17,18,19,20,21]. In particular, they have aroused the interest of several research teams aiming at their use as carriers of nucleic acids [22,23,24,25].

The primary goal of this study was to investigate the formation of complexes of three types of PLL dendrimers (containing additional lysine, arginine, or histidine residues within their structure) with siRNA molecules, to assess the cytotoxicity of dendrimers themselves and the obtained dendriplexes, and finally to evaluate the efficacy of transfection in in vitro-cultured myeloid cell lines.

## 2. Results and Discussion

In recent years, various types of dendrimers have been studied in terms of their use as carriers for oligonucleotides and nucleic acids [26,27,28,29]. It has been shown that PAMAM, phosphorus, and carbosilane dendrimers form stable dendriplexes [29,30] that enable effective transfection and enhancement of the cytotoxic effect of siRNA [31].

**Figure 1:**
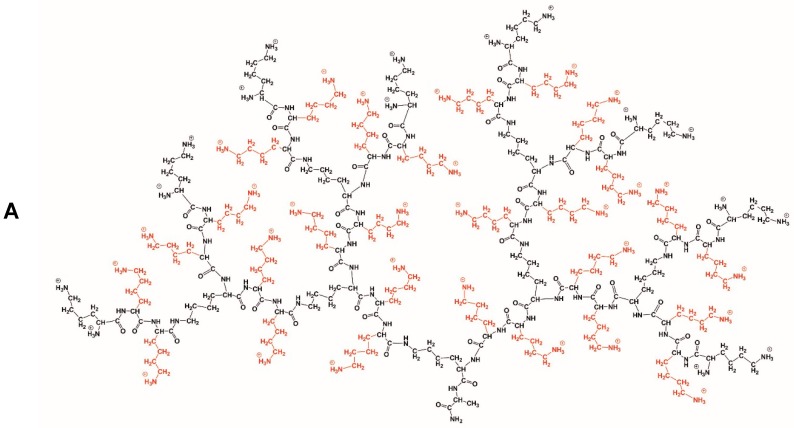

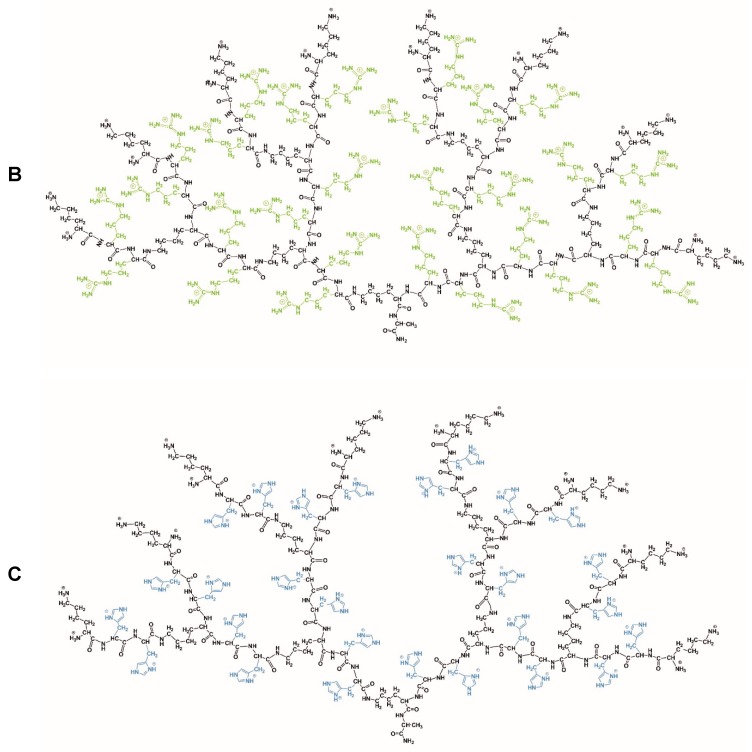
Chemical structures of D3K2 (**A**), D3R2 (**B**), and D3H2 (**C**) dendrimers. The backbone, identical for all dendrimers, is marked in black. The side groups of spacers between each pair of neighboring branching points are marked in red (lysine), green (arginine), and blue (histidine).

For the purpose of drug and gene delivery, PLL dendrimers seem to be the most legitimate choice, primarily due to their biocompatibility, structure flexibility, and positive charge, enabling the formation of non-covalent complexes with nucleic acids and their efficient transport in the bloodstream and into the cell cytoplasm without triggering side effects. In this study, we applied three types of PLL dendrimers (Figure 1): D3K2 (containing two additional lysine residues (Lys-Lys) between each pair of neighboring branching points of the standard lysine dendrimer of the third generation (D3)), D3R2, and D3H2 (containing two additional arginine (Arg-Arg) or histidine (His-His) residues at the same points). Our previous research showed that D3K2 is an excellent carrier of plasmid DNA, with the transfection efficiency comparable to that of the Lipofectamine 2000 [32]. In light of these results, it seemed reasonable to continue the research on the use of PLL dendrimers as carriers for other types of genetic material.

Substitution of lysine residues with different amino acids may bring several benefits to PLL dendrimers considered as a system for gene delivery, changing their flexibility and distribution of charged groups, providing additional interactions with nucleic acids, and affecting cellular uptake levels [24,33,34,35]. Both lysine and arginine are charged aliphatic amino acids with amino groups that are protonated under biological conditions, which may ensure the efficient delivery of nucleic acid into the cells and its endosomal escape. It has been shown that the insertion of Arg residues between inner Lys branching points made the dendrimer’s interior more charged and thus more hydrophilic [34]. What is more, a guanidine moiety of Arg shows high affinity for phosphate groups of DNA, increasing its condensation. On the other hand, His contains an aromatic imidazole group, which can be either cationic or neutral at different pH values, potentially providing additional interactions with nucleic acids and their pH-triggered release [36].

The main goal of this work was the initial characterization of dendrimer:siRNA complexes and evaluation of the in vitro transfection efficiency to determine the optimal conditions for further research.

In the first stage, measurements of fluorescence polarization and changes in the zeta potential of fluorescein-labeled siRNA (siRNA-FITC) under the influence of dendrimers were performed, as well as an evaluation of the hydrodynamic diameter of the obtained complexes. The aim of this step was to determine the optimal molar ratios for complexing siRNA with PLL dendrimers, and the size of the dendriplexes prepared in selected molar ratios.

**Figure 2:**
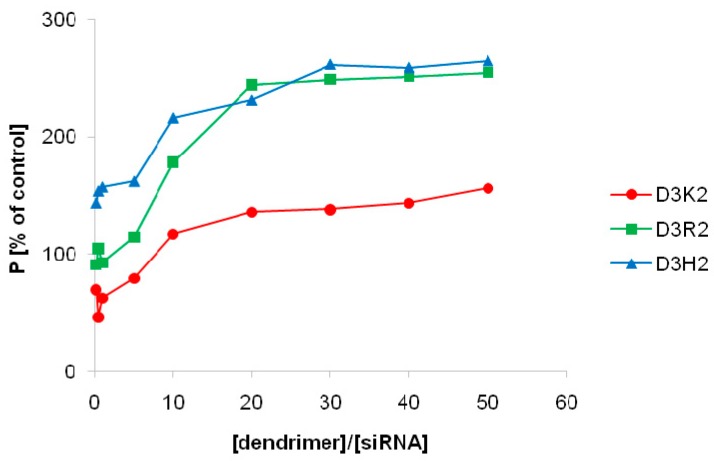
Changes in siRNA-FITC fluorescence polarization as a function of dendrimer:siRNA molar ratio. Results are presented as percentage of control (average ± SD, *n* = 3).

The formation of the dendrimer:siRNA complexes leads to a significant reduction in the movement of siRNA molecules in suspension, which is observed as an increase in the fluorescence polarization of siRNA-FITC (Figure 2). This phenomenon enables the determination of interactions between dendrimers and nucleic acid, and estimation of stoichiometric ratios in which stable complexes are formed.

During the addition of subsequent portions of tested dendrimers to siRNA-FITC solution, a constant increase in fluorescence polarization was observed until reaching the plateau phase, indicating the formation of complexes. A similar observation was made for the complexes of carbosilane dendrimers and oligonucleotides [37,38]. For the D3K2 dendrimer, the increase in fluorescence polarization was much lower compared to D3R2 and D3H2, suggesting that the interactions between the first dendrimer and siRNA are weaker.

The plateau phase was obtained at the following dendrimer:siRNA molar ratios: 20:1 for D3K2 and D3R2, and 30:1 for D3H2 dendrimer. These results suggest that for the complex to form, the siRNA molecule must be surrounded by the studied dendrimers. In addition, this outcome indicates the need to use higher concentrations of PLL dendrimers for the preparation of dendriplexes in comparison to other dendrimers, e.g., carbosilane, for which the optimal dendrimer:siRNA molar ratio was 4:1 [9,26]. It is worth noting, however, that a higher ratio of dendrimer to nucleic acid may allow a dendriplex with a higher positive charge to be obtained, increasing its affinity for cellular membranes.

**Figure 3:**
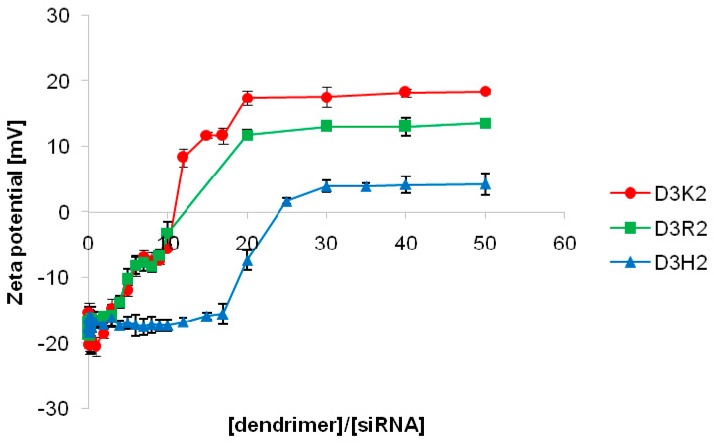
Changes in the zeta potential as a function of the dendrimer:siRNA molar ratio (average ± SD, *n* = 3).

To confirm the stoichiometric ratios of dendrimer:siRNA complexes obtained during the fluorescence polarization measurements, and to assess the surface electrostatic potential of the dendriplexes, zeta potential titration was conducted (Figure 3). Upon the addition of the dendrimers, the initial zeta potential of siRNA-FITC of −14.24 ± 1.59 mV began to rise with the increase of the dendrimer concentration. This trend continued until a plateau was reached at the dendrimer:siRNA molar ratio of 20:1 for D3K2 and D3R2, and 30:1 for the D3H2 dendrimer, which confirmed the stoichiometry of the dendriplex formation determined in the previous stage of study.

The achievement of a plateau, despite the continued addition of the dendrimer, indicates the maximum number of dendrimer molecules attached to one siRNA molecule. Positive zeta potential values have been previously demonstrated for dendriplexes formed by combining siRNA with PAMAM (~10–20 mV), phosphorus (~10–15 mV), or carbosilane dendrimers (~5–30 mV) [26,30,39,40,41], as well as for the complexes of carbosilane dendrimers and oligonucleotides (~5–20 mV) [27]. Here, it was also demonstrated that dendriplexes formed by D3K2 and D3R2 dendrimers have a greater surface positive charge compared to D3H2 dendrimer. This is most likely related to the zeta potential values of the dendrimers themselves (Table 1), and suggests that dendriplexes formed by D3H2 dendrimers may have a lower tendency to interact with the cell membrane, and thus, lower transfection efficacy. On the other hand, a high surface positive charge may increase the cytotoxicity of compounds [10].

Interestingly, given the surface potential of the dendrimers under evaluation, it could be expected that interactions with negatively charged siRNA molecules will be the strongest for the D3K2 dendrimer, which is, however, contradicted by fluorescence polarization studies. This may be partially explained by differences in the flexibility of the studied dendrimers, or additional interactions provided by arginine and histidine residues (either hydrophilic, hydrophobic, or hydrogen bonds) [36], and require further studies.

The hydrodynamic diameter of the dendriplexes formed in the determined optimal molar ratios equaled 841.00 ± 21.95 nm, 300.53 ± 12.16 nm, and 249.67 ± 14.02 nm (dendriplexes formed using D3K2, D3R2, and D3H2 dendrimers, respectively). These results suggest that the cellular uptake of D3K2 dendriplexes may be hindered due to the significant size. However, it has been shown that dendriplexes based on PAMAM and carbosilane dendrimers, characterized by similar sizes, can effectively transport siRNA into the cells [26,27,30,31,42].

The results of this stage of research allowed the ability of cationic PLL dendrimers to form complexes with siRNA to be demonstrated, which can protect it against the action of nucleolytic enzymes and enable the transport of negatively charged nucleic acid molecules through cell membranes.

Next, the cytotoxicity of the dendrimers was evaluated (Table 2). It was shown that the dendrimers exhibit varied cytotoxic activity towards the investigated myeloid cell lines, with D3K2 being the most toxic and D3H2 the least toxic. This outcome is consistent with the zeta potential measurements, indicating the highest positive surface electrostatic potential of D3K2 dendrimer and the lowest in the case of D3H2, suggesting the well-known mechanism of cell death triggered by positively charged dendrimers [43,44]. Due to the observed cytotoxic effects, dendrimers at 1 (D3K2, D3R2) or 1.5 μM (D3H2) concentrations were used for further experiments. These concentrations are required to carry the same concentration of siRNA (0.05 µM) at the optimal dendrimer:siRNA molar ratios.

**Figure 4:**
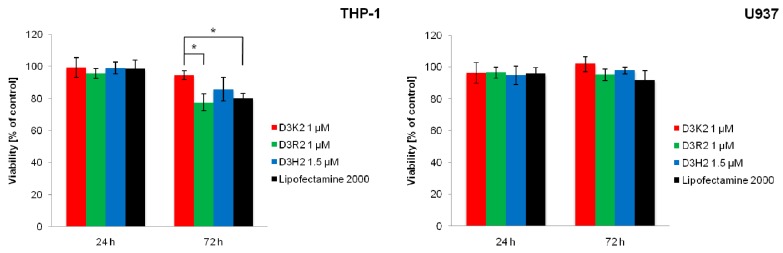
The effect of dendriplexes on the viability of THP-1 and U937 cell lines, presented as a percentage of untreated control (average ± SD, *n* = 3). The dendrimer:siRNA molar ratios equaled 20:1 (D3K2, D3R2) and 30:1 (D3H2). * Statistically significant difference at *p* < 0.05.

The cytotoxicity of dendriplexes after two different incubation times (24 and 72 h) was also determined (Figure 4). The purpose of this step was to compare the toxicity of the obtained dendriplexes with each other and with the cytotoxicity of transfection complexes obtained using the commercially available agent Lipofectamine 2000. This compound, although used as a standard, has been shown to exhibit significant toxicity towards several cell lines, especially during longer incubation times, thus limiting the possibility of its application [45,46].

After 24 h of incubation, the lack of a statistically significant difference between the cytotoxicity of dendriplexes and transfection complex formed using Lipofectamine 2000 was demonstrated for both cell lines. Additionally, no statistically significant difference was found when comparing the dendriplexes with each other. After 72 h of incubation, a statistically significant decrease in the cell viability of the THP-1 cell line transfected with Lipofectamine 2000 was observed, relative to D3K2 dendriplex. In addition, a statistically significant difference was determined between the cytotoxicity of the dendriplex formed by D3R2 (decrease of cell viability to 77.5%) relative to the dendriplex formed by D3K2.

It is worth noting that, despite the differences shown, the dendriplexes and transfection complex formed using Lipofectamine 2000 are characterized by relatively low cytotoxicity. After 24 h of incubation, the viability in all examined cases did not fall below 95%. The highest decrease in viability was observed after 72 h of incubation for the D3R2 dendriplex and the Lipofectamine 2000:siRNA complex; however, in both cases, cell viability was still high (above 70%).

**Figure 5:**
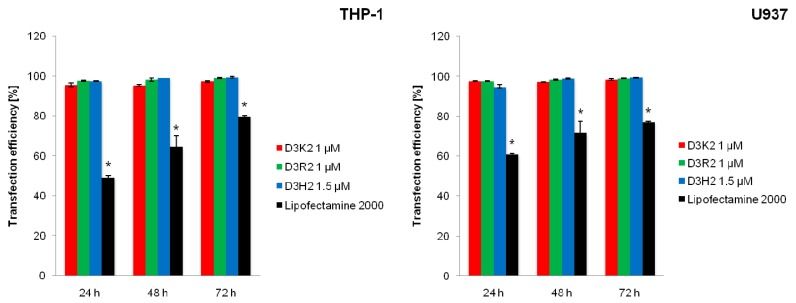
Efficiency of cellular uptake of siRNA-FITC by THP-1 and U937 cells (average ± SD, *n* = 3). The dendrimer:siRNA molar ratios equaled 20:1 (D3K2, D3R2) and 30:1 (D3H2). * Statistically significant difference between dendriplexes and Lipofectamine 2000:siRNA complex at *p* < 0.05.

In the last stage of the study, the efficacy of siRNA cellular uptake was assessed using flow cytometry (Figure 5). Increased cellular uptake of siRNA-FITC molecules was demonstrated for all three PLL dendrimers tested as carriers relative to Lipofectamine 2000. For the dendrimers, the percentage of FITC-positive cells of both cell lines was above 94% regardless of the incubation time. The use of Lipofectamine 2000 as a transfection agent allowed for cellular uptake of siRNA molecules at 49.25% ± 1.05%, 64.6% ± 5.45%, and 79.85% ± 0.35% (THP-1 cell line, 24, 48, and 72 h of incubation, respectively), and 60.85% ± 0.45%, 71.7% ± 5.6%, and 76.9% ± 0.5% (U937 cell line, 24, 48, and 72 h of incubation, respectively).

The obtained results showed that regardless of the incubation time, the level of cellular uptake of dendriplexes is much higher compared to the uptake of complexes formed by Lipofectamine 2000, which correlates well with the results of other studies on the use of PLL dendrimers as carriers for nucleic acids [23,47]. Moreover, despite the differences in the dendrimer–siRNA interactions and dendriplex properties, all tested dendrimers were able to transfect cells with comparable efficiency, reaching ~100%. Similar results were obtained previously by Dzmitruk et al. [31] for phosphorus dendrimers and siRNA with a scrambled sequence; however, the level of transfection was significantly lower for other types of dendrimers (~10% for PAMAM G3, ~60% for PAMAM G4, and ~30% for carbosilane dendrimers). Comparing our results with the transfection efficacy of other types of dendrimers, it can be concluded that PLL dendrimers can serve as an extremely efficient tool for the delivery of siRNA into the cells in vitro.

In summary, based on the conducted experiments, it can be concluded that the PLL dendrimers D3K2, D3R2, and D3H2 are capable of forming complexes with siRNA, and the obtained dendriplexes differ in the strength of dendrimer–siRNA interactions and binding stoichiometry, as well as the electrostatic surface potential and size. The tested dendrimers exhibited varied cytotoxicity relative to the THP-1 and U937 cell lines, with the highest toxic effect exhibited by the dendrimer with the highest surface electrostatic potential. The use of PLL dendrimers made it possible to obtain non-toxic and highly efficient siRNA delivery systems in vitro, increasing the cellular uptake of nucleic acid molecules compared to the commercially available transfection agent.

## 3. Materials and Methods

### 3.1. Dendrimers

PLL dendrimers of the 3rd generation (D3): D3K2 (containing two additional lysine residues (Lys-Lys) between each pair of neighboring branching points of standard lysine dendrimer of the 3rd generation), D3R2 and D3H2 (with two additional arginine (Arg-Arg), or histidine (His-His) residues, respectively, between the same branching points) (Figure 1) were synthesized, purified, and characterized as described previously [33,34,35].

### 3.2. Evaluation of Dendriplex Formation

#### 3.2.1. Fluorescence Polarization Studies

Phosphate-buffered saline (PBS, 10 mM, pH 7.4) solutions containing control siRNA with a scrambled sequence, labeled with fluorescein (FITC) (sc-36869, Santa Cruz Biotechnology, Inc., Heidelberg, Germany) at constant concentration (0.1 μM), were prepared, and their fluorescence was measured. The siRNA-FITC solutions were subsequently titrated with solutions of the tested dendrimers (D3K2, D3R2, or D3H2) at concentrations ranging from 0.002 to 5 μM (in order to achieve dendrimer:siRNA molar ratios ranging from 1:50 to 50:1). The dendrimer:siRNA mixtures were incubated at room temperature for 10 min to ensure the formation of complexes, and then fluorescence anisotropy was measured on a Perkin-Elmer LS-55 spectrofluorometer in a 1-cm path length quartz cuvettes. The excitation and emission wavelengths were 485 and 520 nm, and the excitation and emission slit widths were set to 7 and 5 nm, respectively. The cuvette holder was temperature controlled (25 °C).

#### 3.2.2. Zeta Potential and Hydrodynamic Diameter Measurements

Measurements of the zeta potential and hydrodynamic diameter were performed with the use of Zetasizer Nano ZS (Malvern Instruments Ltd., Malvern, UK). All samples were placed in the low volume sizing cuvettes (ZEN0112, Malvern) for hydrodynamic diameter determination or in the folded capillary cells (DTS 1070, Malvern) for zeta potential measurements and measured at 25 °C. For zeta potential studies, the solutions of studied dendrimers and siRNA-FITC were prepared and the titrations were carried out analogously to fluorescence measurements. Additionally, the zeta potential measurements of free dendrimers in H_2_O and PBS were performed. The hydrodynamic diameter was measured at a dendrimer:siRNA molar ratio of 20:1 for D3K2 and D3G2, and 30:1 for D3H2. The data were analyzed using the Malvern software.

### 3.3. Cell Culture

THP-1 (acute monocytic leukemia) and U937 (histiocytic lymphoma) human cell lines were purchased from ATCC (Manassas, VA, USA) and maintained under standard conditions in RPMI-1640 medium (Gibco, Thermo Fisher Scientific, Waltham, MA, USA) containing 10% fetal bovine serum, penicillin (100 U/mL), and streptomycin (50 µg/mL) (Sigma Aldrich, Taufkirchen, Germany) at 37 °C in an atmosphere of 5% CO_2_. Cells were sub-cultured three times per week.

### 3.4. Preparation of Dendriplexes

The dendriplexes of previously established optimal dendrimer:siRNA molar ratios were prepared by mixing PBS solutions (for cytotoxicity studies) or Opti-MEM medium (Gibco, Thermo Fisher Scientific, Waltham, MA, USA) solutions (for transfection assays) of siRNA-FITC (0.05 μM) and D3K2 (1 μM), D3R2 (1 μM), or D3H2 (1.5 μM) macromolecules. The mixtures were stirred for 10 min at room temperature to ensure the formation of complexes. The obtained dendriplexes were used in subsequent experiments. The control complex of siRNA-FITC (0.05 μM) with Lipofectamine 2000 (Thermo Fisher Scientific, Waltham, MA, USA) was prepared according to the manufacturer’s protocol.

### 3.5. Cytotoxicity Studies

To estimate the cytotoxicity of dendrimers and dendriplexes, the resazurin assay was performed [48]. Cells were seeded into 96-well black plates at a density of 1.5 × 10^4^ cells per well, and treated with increasing concentrations of D3K2, D3R2, and D3H2 for 24 and 72 h at 37 °C in an atmosphere of 5% CO_2_. Following the incubation, resazurin was added to the culture medium to a final concentration of 12.5 μg/mL and the plates were incubated at 37 °C in darkness to allow the conversion of resazurin to resorufin. The fluorescence of the metabolized resazurin was measured after 2 h at 530 nm excitation and 590 nm emission using the PowerWave HT Microplate Spectrophotometer (BioTek, Winooski, VT, USA). The cytotoxicity of dendriplexes and Lipofectamine 2000:siRNA control complex was evaluated in an analogous manner.

### 3.6. Transfection

Cells were seeded into 12-well plates at a density of 1.5 × 10^5^ cells per well, and treated with dendriplexes and Lipofectamine 2000:siRNA control complex for 24, 48, and 72 h at 37 °C in an atmosphere of 5% CO_2_. Following the incubation, the cells were washed with PBS, suspended in fresh medium, and transferred to flow cytometry tubes. The fluorescence was measured using a Becton Dickinson LSR II flow cytometer (BD Biosciences, San Jose, CA, USA). The green fluorescence at 485 nm excitation and 520 nm emission was measured, and the number of FITC-positive cells was expressed as a percentage of the total number of cells in the sample.

### 3.7. Statistics

For statistical analysis, one-way ANOVA followed by post-hoc Tukey’s test was used. In all tests, *p* values < 0.05 were considered to be statistically significant.

## 4. Conclusions

The performed in vitro tests allowed demonstration of the differences in the cytotoxicity of the tested PLL dendrimers, as well as the high biocompatibility of dendriplexes formed by this type of polymer. Most importantly, a very high level of cellular uptake of dendrimer:siRNA complexes was shown, exceeding 90% regardless of the dendrimer structure. These studies constitute the first stage of evaluation of the possibility of the application of the studied dendrimers as carriers for siRNA and will be continued in a wider scope. Only after demonstrating that the transported nucleic acid effectively inhibits expression of the target gene will it be possible to state that PLL dendrimers are an effective method of cell transfection, and may in the future become the basis of modern gene therapy. Additionally, considering the differences in the physicochemical properties of dendriplexes formed by various types of PLL dendrimers, it is reasonable to continue the studies in the field of their characterization, in order to elucidate the role of different amino acids in the structure of the dendrimer in the interactions with siRNA.

## Figures and Tables

**Table 1:** Zeta potential values of PLL dendrimers (average ± SD, *n* = 6).

	H_2_O			PBS		
	D3K2	D3R2	D3H2	D3K2	D3R2	D3H2
zeta potential (mV)	34.35 ± 4.23	23.89 ± 4.57	10.41 ± 2.38	15.55 ± 1.92	14.03 ± 4.27	6.12 ± 1.38

**Table 2:** Cytotoxicity of PLL dendrimers (IC50 values, average ± SD, *n* = 3).

		IC50 (µM)		
		D3K2	D3R2	D3H2
THP-1	24 h	1.68 ± 0.86	26.67 ± 1.48	29.65 ± 3.21
	72 h	0.17 ± 0.07	7.62 ± 2.15	21.83 ± 1.22
U937	24 h	3.53 ± 1.66	8.97 ± 2.37	34.51 ± 2.28
	72 h	2.19 ± 1.26	6.73 ± 1.67	22.23 ± 2.44

## References

[1] Davis M.E., Zuckerman J.E., Choi C.H.J., Seligson D., Tolcher A., Alabi C.A., Yen Y., Heidel J.D., Ribas A. (2010). Evidence of RNAi in humans from systemically administered siRNA via targeted nanoparticles. Nature.

[2] Tatiparti K., Sau S., Kashaw S., Iyer A. (2017). siRNA delivery strategies: A comprehensive review of recent developments. Nanomaterials.

[3] Whitehead K.A., Langer R., Anderson D.G. (2009). Knocking down barriers: Advances in siRNA delivery. Nat. Rev. Drug Discov..

[4] Hickerson R.P., Vlassov A.V., Wang Q., Leake D., Ilves H., Gonzalez-Gonzalez E., Contag C.H., Johnston B.H., Kaspar R.L. (2008). Stability study of unmodified siRNA and relevance to clinical use. Oligonucleotides.

[5] Chen X., Mangala L.S., Rodriguez-Aguayo C., Kong X., Lopez-Berestein G., Sood A.K. (2018). RNA interference-based therapy and its delivery systems. Cancer Metastasis Rev..

[6] Giacca M., Zacchigna S. (2012). Virus-mediated gene delivery for human gene therapy. J. Control. Release.

[7] Xin Y., Huang M., Guo W.W., Huang Q., Zhang L.Z., Jiang G. (2017). Nano-based delivery of RNAi in cancer therapy. Mol. Cancer.

[8] Singha K., Namgung R., Kim W.J. (2011). Polymers in small-interfering RNA delivery. Nucleic Acid Ther..

[9] Biswas S., Torchilin V. (2013). Dendrimers for siRNA delivery. Pharmaceuticals.

[10] Wu J., Huang W., He Z. (2013). Dendrimers as carriers for siRNA delivery and gene silencing: A review. Sci. World J..

[11] Crespo L., Sanclimens G., Pons M., Giralt E., Royo M., Albericio F. (2005). Peptide and amide bond-containing dendrimers. Chem. Rev..

[12] Ohsaki M., Okuda T., Wada A., Hirayama T., Niidome T., Aoyagi H. (2002). In vitro gene transfection using dendritic poly (L-lysine). Bioconjug. Chem..

[13] Klajnert B., Janiszewska J., Urbanczyk-Lipkowska Z., Bryszewska M., Shcharbin D., Labieniec M. (2006). Biological properties of low molecular mass peptide dendrimers. Int. J. Pharm..

[14] Boyd B.J., Kaminskas L.M., Karellas P., Krippner G., Lessene R., Porter C.J. (2006). Cationic poly-L-lysine dendrimers: Pharmacokinetics, biodistribution, and evidence for metabolism and bioresorption after intravenous administration to rats. Mol. Pharm..

[15] Neelov I., Janaszewska A., Klajnert B., Bryszewska M., Makova N.Z., Hicks D., Pearson H.A., Vlasov G.P., Ilyash M.Y., Vasilev D.S. (2013). Molecular properties of lysine dendrimers and their interactions with Aβ-peptides and neuronal cells. Curr. Med. Chem..

[16] Shavykin O.V., Mikhailov I.V., Darinskii A.A., Neelov I.M., Leermakers F.A.M. (2018). Effect of an asymmetry of branching on structural characteristics of dendrimers revealed by Brownian dynamics simulations. Polymer.

[17] Shavykin O.V., Neelov I.M., Darinskii A.A. (2016). Is the manifestation of the local dynamics in the spin–lattice NMR relaxation in dendrimers sensitive to excluded volume interactions?. Phys. Chem. Chem. Phys..

[18] Neelov I., Falkovich S., Markelov D., Paci E., Darinskii A., Tenhu H., Klajnert B., Peng L., Cena V. (2013). Molecular dynamics of lysine dendrimers. Computer simulation and NMR. Dendrimers in Biomedical Applications.

[19] Neelov I.M., Markelov D.A., Falkovich S.G., Ilyash M.Y., Okrugin B.M., Darinskii A.A. (2013). Mathematical simulation of lysine dendrimers: Temperature dependences. Polym. Sci. Ser. C.

[20] Okrugin B.M., Neelov I.M., Leermakers F.A.M., Borisov O.V. (2017). Structure of asymmetrical peptide dendrimers: Insights given by self-consistent field theory. Polymer.

[21] Shavykin O.V., Leermakers F.A., Neelov I.M., Darinskii A.A. (2018). Self-Assembly of Lysine Based Dendritic Surfactants Modeled by the Self-Consistent Field Approach. Langmuir.

[22] Luo K., Li C., Wang G., Nie Y., He B., Wu Y., Gu Z. (2011). Peptide dendrimers as efficient and biocompatible gene delivery vectors: Synthesis and in vitro characterization. J. Control. Release.

[23] Luo K., Li C., Li L., She W., Wang G., Gu Z. (2012). Arginine functionalized peptide dendrimers as potential gene delivery vehicles. Biomaterials.

[24] Rewatkar P.V., Parekh H.S., Parat M.O. (2016). Molecular determinants of the cellular entry of asymmetric peptide dendrimers and role of caveolae. PLoS ONE.

[25] Santos S.S., Gonzaga R.V., Silva J.V., Savino D.F., Prieto D., Shikay J.M., Silva R.S., Paulo L.H.A., Ferreira E.I., Giarolla J. (2017). Peptide dendrimers: Drug/gene delivery and other approaches. Can. J. Chem..

[26] Ionov M., Garaiová Z., Waczulíková I., Wróbel D., Pędziwiatr-Werbicka E., Gómez-Ramirez R., de la Mata F.J., Klajnert B., Hianik T., Bryszewska M. (2012). siRNA carriers based on carbosilane dendrimers affect zeta potential and size of phospholipid vesicles. Biochim. Biophys. Acta Biomembr..

[27] Pedziwiatr-Werbicka E., Shcharbin D., Maly J., Maly M., Zaborski M., Gabara B., Ortega P., dela Mata J.F., Gómez R., Muñoz-Fernandez M.A. (2012). Carbosilane dendrimers are a non-viral delivery system for antisense oligonucleotides: Characterization of dendriplexes. J. Biomed. Nanotechnol..

[28] Serramía M.J., Álvarez S., Fuentes-Paniagua E., Clemente M.I., Sánchez-Nieves J., Gomez R., de la Mata F.J., Muñoz-Fernández M.Á. (2015). In vivo delivery of siRNA to the brain by carbosilane dendrimer. J. Control. Release.

[29] Wrobel D., Kolanowska K., Gajek A., Gomez-Ramirez R., de la Mata F.J., Pedziwiatr-Werbicka E., Klajnert B., Waczulikova I., Bryszewska M. (2014). Interaction of cationic carbosilane dendrimers and their complexes with siRNA with erythrocytes and red blood cell ghosts. Biochim. Biophys. Acta Biomembr..

[30] Ionov M., Lazniewska J., Dzmitruk V., Halets I.V., Loznikova S., Novopashina D.S., Apartsin E.K., Krasheninina O.A., Venyaminova A.G., Milowska K. (2015). Anticancer siRNA cocktails as a novel tool to treat cancer cells. Part (A). Mechanisms of interaction. Int. J. Pharm..

[31] Dzmitruk V., Szulc A., Shcharbin D., Janaszewska A., Shcharbina N., Lazniewska J., Novopashina D., Buyanova M., Ionov M., Klajnert-Maculewicz B. (2015). Anticancer siRNA cocktails as a novel tool to treat cancer cells. Part (B). Efficiency of pharmacological action. Int. J. Pharm..

[32] Gorzkiewicz M., Konopka M., Janaszewska A., Tarasenko I.I., Sheveleva N.N., Gajek A., Neelov I.M., Klajnert-Maculewicz B. (2020). Application of new lysine-based peptide dendrimers D3K2 and D3G2 for gene delivery: Specific cytotoxicity to cancer cells and transfection in vitro. Bioorg. Chem..

[33] Sheveleva N.N., Markelov D.A., Vovk M.A., Mikhailova M.E., Tarasenko I.I., Neelov I.M., Lähderanta E. (2018). NMR studies of excluded volume interactions in peptide dendrimers. Sci. Rep..

[34] Sheveleva N.N., Markelov D.A., Vovk M.A., Mikhailova M.E., Tarasenko I.I., Tolstoy P.M., Neelov I.M., Lähderanta E. (2019). Lysine-based dendrimer with double arginine residues. RSC Adv..

[35] Sheveleva N.N., Markelov D.A., Vovk M.A., Tarasenko I.I., Mikhailova M.E., Ilyash M.Y., Neelov I.M., Lahderanta E. (2019). Stable Deuterium Labeling of Histidine-Rich Lysine-Based Dendrimers. Molecules.

[36] Yang J., Zhang Q., Chang H., Cheng Y. (2015). Surface-engineered dendrimers in gene delivery. Chem. Rev..

[37] Chonco L., Bermejo-Martín J.F., Ortega P., Shcharbin D., Pedziwiatr E., Klajnert B., de la Mata F.J., Eritja R., Gómez R., Bryszewska M. (2007). Water-soluble carbosilane dendrimers protect phosphorothioate oligonucleotides from binding to serum proteins. Org. Biomol. Chem..

[38] Shcharbin D., Pedziwiatr E., Chonco L., Bermejo-Martín J.F., Ortega P., de la Mata F.J., Eritja R., Gómez R., Klajnert B., Bryszewska M. (2007). Analysis of interaction between dendriplexes and bovine serum albumin. Biomacromolecules.

[39] Shcharbin D., Pedziwiatr E., Nowacka O., Kumarb M., Zaborski M., Ortega P., de la Mata F.J., Gómez R., Muñoz-Fernandez M.A., Bryszewska M. (2011). Carbosilane dendrimers NN8 and NN16 form a stable complex with siGAG1. Colloids Surf. B Biointerfaces.

[40] Ferenc M., Pedziwiatr-Werbicka E., Nowak K., Klajnert B., Majoral J.P., Bryszewska M. (2013). Phosphorus dendrimers as carriers of siRNA—Characterisation of dendriplexes. Molecules.

[41] Nam J.P., Nam K., Jung S., Nah J.W., Kim S.W. (2015). Evaluation of dendrimer type bio-reducible polymer as a siRNA delivery carrier for cancer therapy. J. Control. Release.

[42] Leiro V., Duque Santos S., Paula Pego A. (2017). Delivering siRNA with Dendrimers: In Vivo Applications. Curr. Gene Ther..

[43] Thomas T.P., Majoros I., Kotlyar A., Mullen D., Banaszak Holl M.M., Baker J.R. (2009). Cationic poly (amidoamine) dendrimer induces lysosomal apoptotic pathway at therapeutically relevant concentrations. Biomacromolecules.

[44] Ziemba B., Halets I., Shcharbin D., Appelhans D., Voit B., Pieszynski I., Bryszewska M., Klajnert B. (2012). Influence of fourth generation poly (propyleneimine) dendrimers on blood cells. J. Biomed. Mater. Res. Part A.

[45] Wang T., Larcher L., Ma L., Veedu R. (2018). Systematic screening of commonly used commercial transfection reagents towards efficient transfection of single-stranded oligonucleotides. Molecules.

[46] Neuhaus B., Tosun B., Rotan O., Frede A., Westendorf A.M., Epple M. (2016). Nanoparticles as transfection agents: A comprehensive study with ten different cell lines. RSC Adv..

[47] Okuda T., Kidoaki S., Ohsaki M., Koyama Y., Yoshikawa K., Niidome T., Aoyagi H. (2003). Time-dependent complex formation of dendritic poly (L-lysine) with plasmid DNA and correlation with in vitro transfection efficiencies. Org. Biomol. Chem..

[48] O’brien J., Wilson I., Orton T., Pognan F. (2000). Investigation of the Alamar Blue (resazurin) fluorescent dye for the assessment of mammalian cell cytotoxicity. Eur. J. Biochem..

